# Video-Based Actigraphy for Monitoring Wake and Sleep in Healthy Infants: A Laboratory Study

**DOI:** 10.3390/s19051075

**Published:** 2019-03-03

**Authors:** Xi Long, Renée Otte, Eric van der Sanden, Jan Werth, Tao Tan

**Affiliations:** 1 Department of Family Care Solutions, Philips Research, 5656 AE Eindhoven, The Netherlands; renee.otte@philips.com (R.O.); eric.van.der.sanden@philips.com (E.v.d.S.); 2 Department of Electrical Engineering, Eindhoven University of Technology, 5612 AP Eindhoven, The Netherlands; jan.werth@philips.com; 3 Department of Mathematics and Computer Science, Eindhoven University of Technology, 5612 AP Eindhoven, The Netherlands; tao.tan911@gmail.com

**Keywords:** Infant monitoring, wake-sleep pattern, infrared camera, video-based actigraphy, classification

## Abstract

Prolonged monitoring of infant sleep is paramount for parents and healthcare professionals for interpreting and evaluating infants’ sleep quality. Wake-sleep patterns are often studied to assess this. Video cameras have received a lot of attention in infant sleep monitoring because they are unobtrusive and easy to use at home. In this paper, we propose a method using motion data detected from infrared video frames (video-based actigraphy) to identify wake and sleep states. The motion, mostly caused by infant body movement, is known to be substantially associated with infant wake and sleep states. Two features were calculated from the video-based actigraphy, and a Bayesian-based linear discriminant classification model was employed to classify the two states. Leave-one-subject-out cross validation was performed to validate our proposed wake and sleep classification model. From a total of 11.6 h of infrared video recordings of 10 healthy term infants in a laboratory pilot study, we achieved a reliable classification performance with a Cohen’s kappa coefficient of 0.733 ± 0.204 (mean ± standard deviation) and an overall accuracy of 92.0% ± 4.6%.

## 1. Introduction

During the first year of life, the average infant is asleep for a greater part of the day than they are awake [1], making sleep one of the most important activities for their brains [2]. Essential developmental processes take place during sleep, and problems with sleeping, therefore, may inhibit optimal development [3]. Sleep tracking and assessment can offer valuable information on an infant’s mental and physical development, not only for healthcare professionals, but also for parents [4,5]. This information, for instance, could enable tailored coaching for parents and help improve an infant’s sleep quality [6,7]. 

The most common way to monitor infant sleep is by polysomnography (PSG) [8], including multiple sensor modalities, such as electroencephalography, electrooculography, electromyography, electrocardiography, respiration, and others. A drawback of this method is that it requires obtrusive contact sensing on the body of an infant, which is undesirable in sick or fragile infants, and impractical in a home situation. Moreover, for reliable measures of wake-sleep patterns, prolonged monitoring over multiple nights is recommended [9,10], increasing the need for unobtrusive monitoring. 

Over the past decades, a number of unobtrusive or minimally obtrusive techniques have been developed for objective infant (sleep) monitoring, such as actigraphy [9], capacitive sensing [11], ballistocardiography [12], photoplethysmography [13,14], laser Doppler vibrometers [15], and (near) infrared (IR) or thermal video [16,17]. These techniques have been summarized in previous systematic reviews [18,19]. In particular, video-based monitoring appears to be a promising technique, since, in contrast to wearables, it is entirely unobtrusive, does not require mounting/unmounting, and does not need battery charging, making it convenient and easy to use in real home situations. Video-based approaches are capable of capturing body movements that are highly correlated with infant wake/sleep state [20]. A large number of studies that employed wrist or ankle actigraphy to measure gross body movements have achieved good performance in wake/sleep detection both for adults and children [21,22]. Heinrich et al. [23] demonstrated that video-based solutions are able to replace on-body actigraphic sensors for the application of sleep monitoring and analysis. Furthermore, video-based approaches potentially allow for the measurement of vital signs (e.g., heart rate and breathing rate) [17,24], which are of importance for automatically identifying infant sleep stages (active/quiet sleep).

In this paper, we describe a new method for reliably classifying infant wake and sleep states using motions derived from IR video frames (i.e., video-based actigraphy). The use of an IR video camera allows for the monitoring of infants in a dark environment without visible light. As this was an explorative, proof-of-concept study on the feasibility of using video-based actigraphy for classifying infant wake and sleep states, we started with a small pilot population of healthy infants.

## 2. Methods

### 2.1. Subjects and Data

Data from 10 healthy infants aged 5 months on average (range 3–9 months) were included in this study. The infants’ parents had been recruited through word-of-mouth, flyers aimed at young parents living close to Tilburg University, the website of the Tilburg University BabyLab, and meetings for expectant couples in the maternity ward of St. Elisabeth Hospital in Tilburg, The Netherlands. The exclusion criteria were health problems or problems related to feeding, sleeping, or development. 

Before consenting to have their baby participate, all steps of the study were explained in full to the infant participants’ parents. Both parents signed an informed consent form if they agreed to let their baby participate in the study. The study protocol was approved by both the Internal Ethics Committee for Biomedical Experiments of Philips Research Eindhoven, and the Psychological Ethical Test Committee of Tilburg University, The Netherlands. The study was performed in conformity with the declaration of Helsinki. As the study was observational in nature, no additional approval from an external ethics board was required—the Dutch law on medical scientific research with human beings (WMO) was not applicable.

Video recordings were obtained from an IR camera placed in a “look-down” view above an infant bed placed in the BabyLab at Tilburg University. The whole mattress of the bed was visible. For each infant video, data with an average duration of 1.16 ± 0.43 (mean ± standard deviation) hours was included, resulting in a total of 11.6 h used for the analyses. Synchronized to the IR videos sleep stages were scored as wake, rapid eye movement (REM) sleep, and non-REM (NREM) sleep including N1, N2, and N3 stages by a trained sleep specialist for non-overlapping epochs that lasted 30 s, adhering to the rules of the American Academy of Sleep Medicine [25] and those specifically for infant sleep scoring by Grigg-Damberger et al. [8]. The scoring was based on multichannel PSG signals collected using a commercially available PSG system (Vitaport3, TEMEC Instruments B.V., Heerlen, The Netherlands).

Since this study only focused on automatic classification of wake and sleep states, all annotated REM and N1–N3 sleep epochs were grouped into a single sleep state. Wake and sleep epochs accounted for 28.0% and 71.3% of the data, respectively. “Unable-to-score” epochs made up 0.7% of the data, and pertained to epochs for which the signal quality was poor (caused by poor or no connectivity of the PSG electrodes). The PSG annotations served as the golden standard for automated video-based classification of infant wake/sleep states.

### 2.2. Video-Based Actigraphy

Estimated with an IR camera, video-based actigraphy (i.e., a recording of movements by means of a camera) can be used with high accuracy to assess whether or not an infant is in bed; in a recent study, 96.9% accuracy was achieved [26]. Also, since infants’ movements yield information on their behavioral state [27], the technique can be applied for discrimination of these behavioral states. In the current work, we focused on infant wake and sleep classification.

**Figure 1:**
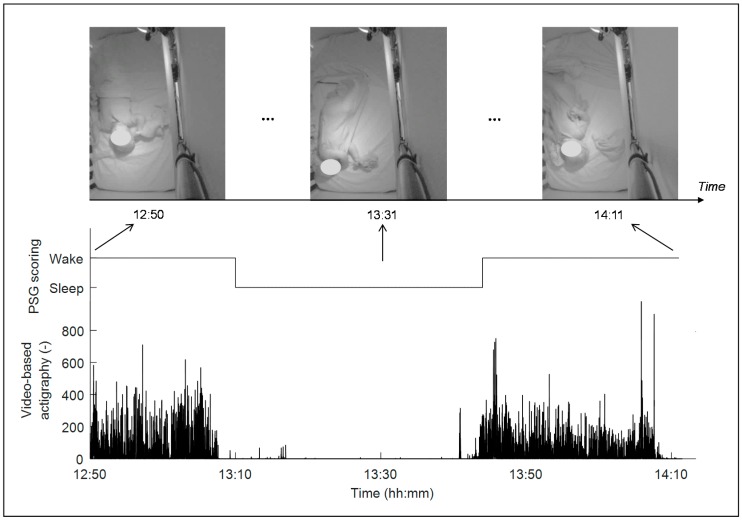
Example of video frames in a 1.35 h recording, and the estimated video-based actigraphy and wake/sleep state. The sampling rate of the video and the video-based actigraphy is 10 Hz. The face of the infant is blurred for privacy purposes.

To obtain video-based actigraphy, we employed a spatiotemporal-based recursive search (RS) motion detection algorithm to quantify motions from IR videos [23,28,29]. Compared with other motion detection algorithms, such as optical flow, this RS algorithm has been shown to be robust to scene changes, or more specifically in this case, to changes due to illumination, which can be eliminated. The IR video recording (at 376 × 480 pixels) and its corresponding raw RS motion estimates had a frame rate of 10 Hz. Figure 1 shows an example including three IR video frames within a 1.35 h video recording from an infant, and the corresponding video-based actigraphy derivation, which seems highly correlated to the infant’s wake/sleep state. Larger values of the estimated video-based actigraphy correspond to larger or more body movements.

### 2.3. Feature Extraction and Classification Model

Two features were extracted on a 30 s basis from the raw video-based actigraphy signal. First, we calculated the mean activity count (mACT, i.e., count of non-zero motions) over video frames for each epoch, similar to wrist actigraphy, which is intensively utilized in wake/sleep identification [30]. A disadvantage of mACT is that it is challenging to identify wakefulness when body movement is reduced. Therefore, we characterized the “‘possibility” of being asleep (pSLP) before and after a very high activity level [31]. This was done by quantifying the logarithm of the time difference between each epoch and its nearest epoch with a large amount of body movements (corresponding to a large mACT value). It was then smoothed through a moving average, with an experimentally optimized window of 10 min. For each recording, an epoch with an mACT value larger than the 95th percentile of the mACT values over the entire recording was considered to have a high activity level. pSLP is expected to correctly identify some “low movement” wake epochs when they are very close to wake epochs with a lot of body movements (i.e., a high activity level). The hypothesis, thus, is that epochs with little movement that are close to those with a high level of activity (and thereby with a smaller time difference) are more likely to correspond to the infant being awake than to the infant being asleep. 

**Figure 2:**
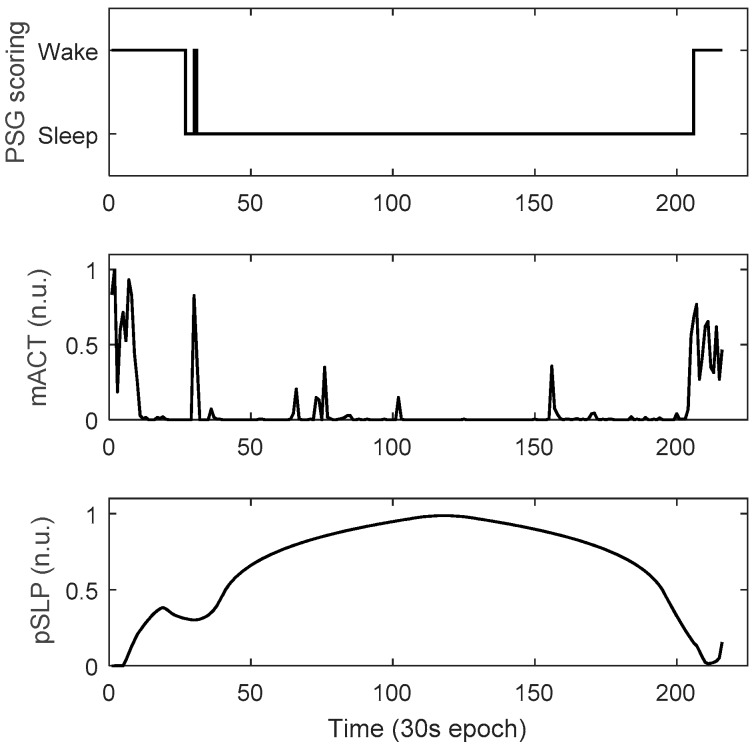
Example of 30 s-based wake and sleep annotations (polysomnography—PSG) and the corresponding feature values of the two features, mean activity count (mACT) and the “possibility” of being asleep (pSLP) (normalized unit: n.u.), from a recording of 216 epochs.

To diminish the “global” variability between infants conveyed by video-based actigraphy, we needed to normalize the features. For each feature and each infant this was done with a Min–Max method to rescale the feature values within a range between 0 and 1. As can be seen in Figure 2, the two features mACT and pSLP show a high correlation with the infant wake-sleep pattern. Notably, although almost no body movements were observed during the wake period between the 10th and the 30th epochs (corresponding to low mACT values), the possibility of the infant being awake was high, due to the relatively low feature values of pSLP. This is because, for these wake epochs, the time to the epochs with a high activity level (high mACT) was relatively short.

A Bayesian-based linear discriminant classification model (classifier) was deployed, which has been successfully used in sleep classification in previous studies [21,32,33]. The classifier is based on Bayes decision rules for minimizing the probability of error, i.e., to choose the class that maximizes its posterior probability given an observation (feature vector). Note that wake was set to be the positive class and sleep the negative class in this dataset, and the classifier’s prior probability for both classes was equalized.

### 2.4. Validation and Assessment

For the two video-based actigraphic features statistical analysis (Mann–Whitney *U* test) was performed to examine the difference between all wake and sleep epochs from all infants.

To show the validity of the proposed classification model, we applied a (subject-independent) leave-one-subject-out cross validation (LOOCV). During each round of the LOOCV, we used data from nine infants to train the classifier, and that from the remaining infant to test the classifier. Ten rounds were conducted and the performance was reported by averaging classification results over all rounds.

Commonly used metrics, including accuracy, precision, sensitivity, and specificity, as well as the area under the “receiver operating characteristic” (ROC) curve (AUC) were considered to assess the wake-sleep classification performance. Since the binary class distribution was imbalanced, as the wake epochs accounted for only 28.0%, the chance-compensated metric Cohen’s kappa coefficient was also employed, and this metric has been widely used for evaluating sleep scoring or sleep staging reliability. In this work, the algorithms were implemented in MATLAB R2016b (MathWorks, Natick, MA, USA).

## 3. Results and Discussion

**Figure 3:**
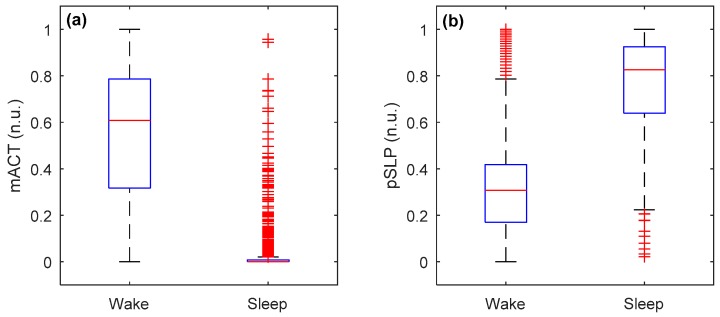
Boxplots of the two features (**a**) mACT and (**b**) pSLP during wake and sleep (of all epochs pooled over all infants) in normalized unit (n.u.), where wake epochs had higher mACT and lower pSLP than sleep epochs (*p* < 0.0001).

Mean and standard deviation of the two proposed features (mACT and pSLP, on a 30 s epoch basis) from video-based actigraphy from all recordings (or infants) during wake and sleep states are shown in Figure 3. A significant difference at *p* < 0.0001 was found between the two states for both features, indicating that they were effective in discriminating between wake and sleep epochs. However, there were still overlaps between wake and sleep states in both features, which would be difficult to correctly identify.

**Figure 4:**
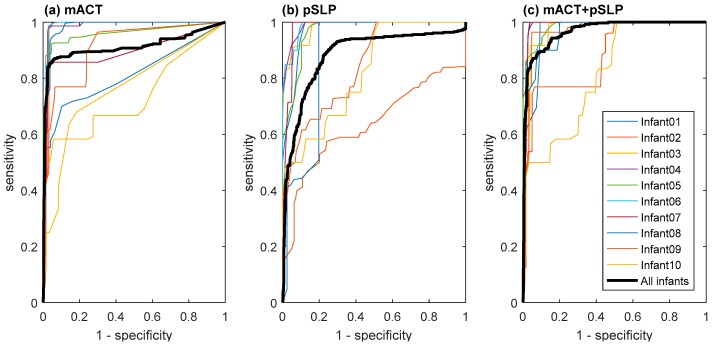
Receiver operating characteristic (ROC) curves of each infant and all epochs pooled over all infants obtained, based on the proposed wake and sleep classification model using three different feature sets (**a**) mACT, (**b**) pSLP, and (**c**) mACT + pSLP, where the area under the curve (AUC) for all infants is 0.902, 0.877, and 0.952.

Table 1 compares the LOOCV wake and sleep classification results using different feature sets, i.e., mACT only, pSLP only, and their combination (mACT + pSLP). The best-performing result for accuracy and Cohen’s kappa is in bold. In addition, the performance comparison in the ROC space can be seen in Figure 4. Combining the two features can largely improve the wake and sleep classification performance, where a substantial agreement with a mean Cohen’s kappa coefficient of 0.733 ± 0.204, and a mean accuracy of 92.0% ± 4.6% across infants was achieved.

Interestingly, the feature pSLP was able to help reduce the number of false negatives (i.e., number of wake epochs incorrectly classified as sleep epochs) and thus increase the precision and sensitivity. After a closer look, we found that these corrected false negatives mostly corresponded to wake epochs with little or even no infant body movements that were very close to epochs with a high mACT value (i.e., a lot of body movements). This can be further observed by comparing the ROC curves in Figure 4. For example, by changing the classifier’s decision-making threshold to have a specificity of ~55%, the sensitivity can go up to 100%, corresponding to zero false negatives when including pSLP, while it is ~90% when excluding pSLP.

**Figure 5:**
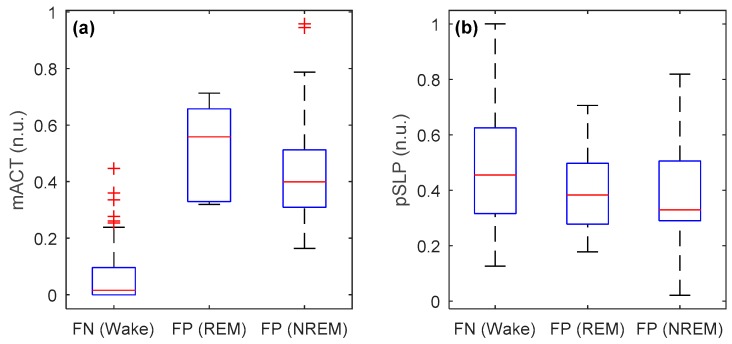
Boxplots of misclassifications for the two features (**a**) mACT and (**b**) pSLP in normalized unit (n.u.) where false negatives (FN) are the number of wake epochs misclassified as sleep epochs (FN = 71) and false positives (FP) are the number of sleep epochs incorrectly classified as wake epochs (FP = 7 for rapid eye movement (REM) sleep, FP = 28 for non-REM (NREM) sleep).

The classification accuracy and kappa for each infant are presented in Table 2. We noticed that the kappa value for Infant10 is very low (0.220). Video inspection showed this to be because this baby was taken out of bed for around one-fifth of the video recording, during which the PSG was still available with wires connected, whereas the infant was not visible through the video camera. For this infant, when discarding the out-of-bed period, the kappa and accuracy increased to 0.642 and 94.8%, respectively. Nevertheless, this is a drawback of using video camera for infant sleep monitoring: it requires the infant to be in the bed all the time. In a recent work [26], we have shown that it is possible to accurately identify whether an infant is in bed or out of bed (with an accuracy of 96.9%), so that out-of-bed periods can be removed before doing wake and sleep classification. Apart from that, the performance variability across infants remains relatively high (with the kappa ranging from 0.629 to 0.939). One explanation is that for some recordings external disturbances (e.g., from parental activity) also contributed to the motion captured by the videos, likely leading to misclassification of some sleep epochs as wake (false positives). In addition, it could be caused by inter-subject variability in infant weight, age, sleep pattern (in particular, in this dataset with only short recordings), arousals, subtle body movements such as jerks and scratches, and even autonomic regulation [34,35]. We speculate that some misclassifications were between wake and REM sleep with body movements, and between sleep and “motionless wake” without, or with fewer, body movements. The feature values of false negative (FN) and false positive (FP) misclassifications are compared in Figure 5. It can be clearly seen that the mACT values of FN (wake) epochs were close to zero, much lower than those of FP epochs and other wake epochs (see Figure 3). On the other hand, the FP (sleep) epochs, in particular during REM sleep, had very high mACT values. With regard to pSLP, on average, FN epochs had even slightly higher values than FP epochs for both REM and NREM sleep. It would be very difficult to correctly identify these misclassifications using video-based actigraphy only. Therefore, besides physical activity, vital signs such as respiration and heart rate (and heart rate variability), that are able to characterize autonomic nervous activity, are then required for further improving the classification.

In general, the results of our wake and sleep classification model are better than those reported in literature using accelerometer-based actigraphy [20,30]. This indicates that an IR video camera is a feasible instrument to reliably monitor wake and sleep states of healthy term infants in bed. However, the feasibility for infants with sleep disorders should be validated in the future.

It is important to note that this was a laboratory study, where the monitoring took place in a “controlled” environment with a fixed camera placement and relatively stable lighting conditions for all infants. Additionally, only a small dataset was used including 10 infants with an average of 1.15 h recording per infant. In a real application of infant sleep monitoring at home, we would encounter challenges such as various camera placements with different angles and distances to the infant’s bed, changes in brightness or darkness in the room, video occlusion, and other disturbances (e.g., parental activity). These would likely cause a decline in classification performance. Therefore, a home study in a free-living setting with a larger dataset, including more infants, longer and multiple-day recordings that can capture daily wake-sleep rhythms, should be conducted in the future in order to validate the method proposed in this paper. Advanced video processing and machine learning algorithms to deal with these challenges merit further investigation.

Furthermore, as for this proof-of-point study, we included only healthy infants. The accuracy regarding sick or preterm infants and those with sleep disorders remains unclear. Further research is needed to validate the methodology for these clinical populations. In addition to validation, future work could focus on the type and quality of movements during in bed periods to help in detection, monitoring, and classification of sleep-related movement disorders, such as restless legs syndrome, periodic limb movement, night terrors/nightmares, head banding, excessive somnolence, insomnia, jet lag syndrome, and sleep-disordered breathing. For movement-related disorders, advanced video processing and pattern/texture analysis methods—rather than general motion detection—would potentially be possible to capture the specific type and quality of disorder-related movements. For infants with sleep-disordered breathing such as sleep apnea, as mentioned in the introduction, cardiac and respiratory information could be detected from videos [24]. Combining video-based cardiorespiratory and movement information for wake and sleep classification warrants further research in sleep apnea subjects.

## 4. Conclusions

This work experimentally validated the feasibility of automatically classifying wake and sleep states in healthy term infants using an infrared video camera, which is considered to be an unobtrusive (or contactless) method of monitoring and assessing infant sleep. From infrared video frames, video-based actigraphy was estimated, from which two discriminative features were extracted. A Bayesian linear discriminant classification was used. A small set of data was collected in a laboratory study, including video recordings of 11.6 h from 10 infants. Leave-one-subject-out cross validation revealed a substantial agreement with a Cohen’s kappa coefficient of 0.733 ± 0.204 (mean ± standard deviation across infants), compared with polysomnography-based human scoring in wake and sleep classification. Further studies with larger datasets collected in free-living environments at home, and with clinical populations, are necessary in the future.

## Figures and Tables

**Table 1:** Comparison of wake and sleep classification results (leave-one-subject-out cross validation—LOOCV) with video-based actigraphy.

Metrics ^1^		Feature Set	
	mACT	pSLP	mACT + pSLP
True positive (TP)	304	307	318
False positive (FP)	36	143	35
False negative (FN)	85	82	71
True negative (TN)	954	847	955
Precision	82.4% ± 18.3%	61.0% ± 22.7%	82.3% ± 16.6%
Sensitivity	71.9% ± 24.4%	82.8% ± 18.9%	77.4% ± 22.7%
Specificity	95.9% ± 2.6%	84.8% ± 6.5%	95.8% ± 2.6%
Accuracy	91.3% ± 4.7%	82.0% ± 7.9%	**92.0% ± 4.6%**
Cohen’s kappa	0.701 ± 0.227	0.544 ± 0.187	**0.733 ± 0.204**

^1^ For confusion matrix elements, results pooled over all epochs from all infants are presented; for the other metrics, mean ± standard deviation results across infants are presented. The highest accuracy and Cohen’s kappa are in bold.

**Table 2:** Wake and sleep classification results (LOOCV) for each infant.

Infant	Epoch Number	% Wake Epochs	Accuracy	Cohen’s kappa
01	216	17.6%	91.7%	0.664
02	120	46.7%	90.8%	0.816
03	118	10.2%	93.2%	0.629
04	237	30.8%	93.3%	0.837
05	163	58.3%	91.4%	0.826
06	93	21.5%	97.9%	0.939
07	123	5.7%	98.4%	0.866
08	131	38.2%	91.6%	0.817
09	106	24.5%	89.6%	0.716
10	72	16.7%	81.9%	0.220
